# Association of imprinting status of IGF2 with the pathophysiology of intrauterine growth retardation in rats

**DOI:** 10.1186/1687-9856-2013-S1-O45

**Published:** 2013-10-03

**Authors:** Xiuyun Zhou, Xiaoping Luo

**Affiliations:** 1 Departments of Pediatrics, Tongji Hospital, Tongji Medical College, Huazhong University of Science and Technology, Wuhan 430030, China

## 

Insulin like growth factor 2 (IGF2) is a crucial imprinting gene which prompting placenta development and fetal growth. It demonstrates parent-of-origin-specific allelic expression that is epigenetically regulated by differential methylation of IGF2/H19 DMR. We aim to explore if abnormal IGF2 imprinting status that regulated by altered DNA methylation of IGF2/H19 DMR affects IGF2 expression leading to the occurrence of IUGR.

First we established an IUGR model of rat at two different stages(19-d gestational age and 1-day-old) by maternal nutrition restriction, and the rat tissues in the control group and the IUGR group were collected which were divided into four subgroups(N=6~8, each subgroup): Subgroup 1 and Subgroup 2 was the placenta and liver tissue of fetuses with 19-d gestational age, respectively, Subgroup 3 and Subgroup 4 was the liver tissue and skeletal muscle tissue in the posterior limbs of 1-day-old rats, respectively. Then the mRNA and protein levels of IGF2 were tested in the four subgroups of the two groups. In each subgroup, pyrosequencing was used to test the DNA methylation level of imprinting control region IGF2/H19 DMR. We observed that the expression levels of IGF2 in the Subgroup1, Subgroup 2, Subgroup 3 were decreased significantly compared with the control group, but there were no obvious differences in the Subgroup 4 of the two groups. The pyrosequencing showed no significant differences in methylation levels of the CpG dinucleotides in IGF2/H19 DMR between the two groups. What’s more, the methylation ratio didn't change versus age, and maintained the same from fetus till adult, and it also stayed the same among different tissue types except for placenta which had a lower methylation fraction than the other tissues. We concluded that the expression of IGF2 was down-regulated in the IUGR rats, which could play an important role in the pathogenesis of IUGR. However, the down regulation was not always the result of the aberrant imprinting status of the imprinting control region H19 DMR, as there wasn't a clear association between the two. IGF2 could be down regulated through other pathways, such as histone modification, or altered imprinting of another control region, to mediate the development of IUGR.

